# Prospective Multicenter Validation of Androgen Receptor Splice Variant 7 and Hormone Therapy Resistance in High-Risk Castration-Resistant Prostate Cancer: The PROPHECY Study

**DOI:** 10.1200/JCO.18.01731

**Published:** 2019-03-13

**Authors:** Andrew J. Armstrong, Susan Halabi, Jun Luo, David M. Nanus, Paraskevi Giannakakou, Russell Z. Szmulewitz, Daniel C. Danila, Patrick Healy, Monika Anand, Colin J. Rothwell, Julia Rasmussen, Blair Thornburg, William R. Berry, Rhonda S. Wilder, Changxue Lu, Yan Chen, John L. Silberstein, Gabor Kemeny, Giuseppe Galletti, Jason A. Somarelli, Santosh Gupta, Simon G. Gregory, Howard I. Scher, Ryan Dittamore, Scott T. Tagawa, Emmanuel S. Antonarakis, Daniel J. George

**Affiliations:** ^1^ Duke University, Durham, NC; ^2^ Johns Hopkins University, Baltimore, MD; ^3^ Weill Cornell Medical College, New York, NY; ^4^ University of Chicago, Chicago, IL; ^5^ Memorial Sloan Kettering Cancer Center, New York, NY; ^6^ Epic Sciences, San Diego, CA

## Abstract

**PURPOSE:**

Androgen receptor splice variant 7 (AR-V7) results in a truncated receptor, which leads to ligand-independent constitutive activation that is not inhibited by anti-androgen therapies, including abiraterone or enzalutamide. Given that previous reports suggested that circulating tumor cell (CTC) AR-V7 detection is a poor prognostic indicator for the clinical efficacy of secondary hormone therapies, we conducted a prospective multicenter validation study.

**PATIENTS AND METHODS:**

PROPHECY (ClinicalTrials.gov identifier: NCT02269982) is a multicenter, prospective-blinded study of men with high-risk mCRPC starting abiraterone acetate or enzalutamide treatment. The primary objective was to validate the prognostic significance of baseline CTC AR-V7 on the basis of radiographic or clinical progression free-survival (PFS) by using the Johns Hopkins University modified-AdnaTest CTC AR-V7 mRNA assay and the Epic Sciences CTC nuclear-specific AR-V7 protein assay. Overall survival (OS) and prostate-specific antigen responses were secondary end points.

**RESULTS:**

We enrolled 118 men with mCRPC who were starting abiraterone or enzalutamide treatment. AR-V7 detection by both the Johns Hopkins and Epic AR-V7 assays was independently associated with shorter PFS (hazard ratio, 1.9 [95% CI, 1.1 to 3.3; *P* = .032] and 2.4 [95% CI, 1.1 to 5.1; *P* = .020], respectively) and OS (hazard ratio, 4.2 [95% CI, 2.1 to 8.5] and 3.5 [95% CI, 1.6 to 8.1], respectively) after adjusting for CTC number and clinical prognostic factors. Men with AR-V7–positive mCRPC had fewer confirmed prostate-specific antigen responses (0% to 11%) or soft tissue responses (0% to 6%). The observed percentage agreement between the two AR-V7 assays was 82%.

**CONCLUSION:**

Detection of AR-V7 in CTCs by two blood-based assays is independently associated with shorter PFS and OS with abiraterone or enzalutamide, and such men with mCRPC should be offered alternative treatments.

## INTRODUCTION

Men with metastatic castration-resistant prostate cancer (mCRPC) have improved survival when treated with the androgen receptor (AR) inhibitors enzalutamide or abiraterone.^1,2^ However, in men with poor-risk clinical features or prior exposure to one of these agents, response rates are low, and progression-free survival (PFS) and overall survival (OS) times are short. Moreover, cross-resistance is common,^3-5^ and clinical features are unable to predict cross-resistance. Thus, predictive biomarkers are urgently needed to optimize treatment selection.

In many men with mCRPC, the AR pathway remains active despite testicular androgen suppression through cancer-specific AR upregulation, mutation, and paracrine/autocrine androgen synthesis.^6-8^ In addition, constitutive activation through the expression of AR splice isoforms that lack the androgen ligand-binding domain contributes to resistance.^9-12^ Detection of the splice isoform AR-V7 in circulating tumor cells (CTCs) has been strongly associated with abiraterone or enzalutamide resistance^13-15^ but was compatible with responsiveness to taxane chemotherapy.^14-16^ These data suggest that Androgen receptor splice variant 7 (AR-V7) assays may provide predictive utility to guide treatment decisions; however, prospective, multicenter validation is needed. We report the results of PROPHECY (ClinicalTrials.gov identifier: NCT02269982), an independent, multicenter, prospective-blinded validation study of two CTC AR-V7 assays in predicting PFS and OS with abiraterone or enzalutamide in men with mCRPC.

## PATIENTS AND METHODS

### Patients

We prospectively enrolled at five clinical sites men with progressive, high-risk mCRPC initiating standard-of-care treatment with enzalutamide or abiraterone. Prior exposure to enzalutamide or abiraterone was permitted for men who were planning to receive the alternative agent. The Data Supplement discusses the full eligibility criteria and definitions of high-risk disease, which required two or more poor prognosis clinical factors^17,18^ (Table 1). All patients provided written informed consent. The study was approved by institutional review boards of all participating centers within the Department of Defense Prostate Cancer Clinical Trial Consortium,^19^ with Duke University as the lead coordinating center.

**TABLE 1.**
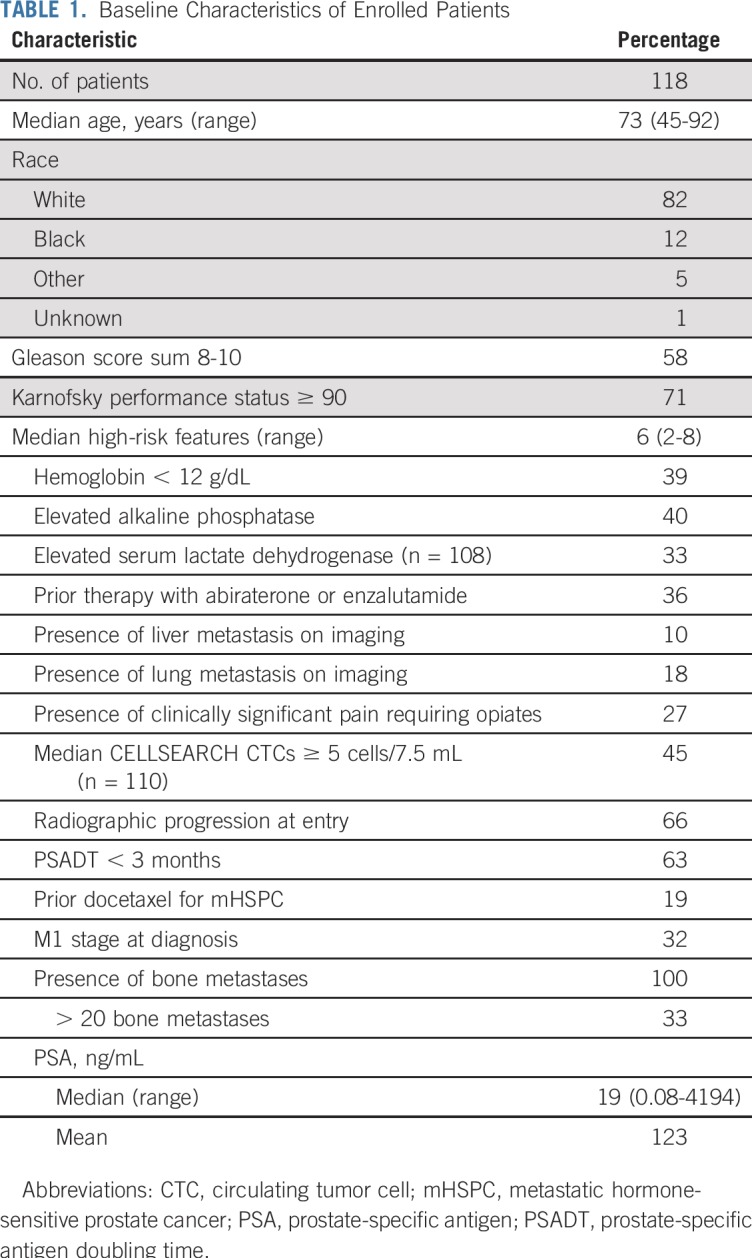
Baseline Characteristics of Enrolled Patients

### Study Design and Assessments

This prospective, multicenter study evaluated the ability of baseline (pretreatment) AR-V7 status in CTCs to predict treatment outcomes with abiraterone or enzalutamide. All the authors vouch for the completeness and integrity of the data and for the fidelity of the study to the clinical protocol (Data Supplement). Peripheral blood samples for analysis of CTCs were obtained from eligible patients at prespecified time points: baseline and at the time of clinical, radiographic, or biochemical progression. CELLSEARCH (Menarini Silicon Biosystems, Bryn Athyn, PA) CTC enumeration was performed at these time points on all patients and processed in a College of American Pathologists/Clinical Laboratory Improvement Amendments–approved central laboratory.^17,20^ Treatment selection was at the discretion of the treating physician without awareness of AR-V7 status. Laboratory investigators were blinded to the clinical information and patient outcomes. All data sets were separately sent to the study statistician (S.H.) who unblinded the data after the database was locked.

### Analysis of CTCs

CTCs were analyzed in two central laboratories, each blinded to the results of the other. CTC identification by the Epic Sciences (Epic; San Diego, CA) CTC nuclear-specific AR-V7 protein assay and CTC heterogeneity evaluations were performed as described previously.^14,21,22^ The Johns Hopkins University (JHU; Baltimore, MD) modified-AdnaTest CTC AR-V7 mRNA assay was performed as previously described using validated methods.^13,23-25^ Established standard operating procedures for sample collection, overnight shipping, processing, and analysis were followed by study sites and the central laboratory at JHU.^13,23,24^ The Data Supplement details additional methods, including CTC heterogeneity criteria using the Shannon index.^22^

### Clinical Outcomes

The primary end point was PFS, defined from date of registration to clinical/radiographic progression or death, whichever occurred first. Radiographic progression was assessed using Prostate Cancer Working Group 2 soft tissue and bone scan criteria.^26^ Clinical progression was defined by death, pain, or other symptomatic progression; initiation of new systemic therapy; or a skeletal-related event. Secondary clinical end points included confirmed 50% or greater prostate-specific antigen (PSA) declines, radiographic response per RECIST version 1.1,^27^ and OS. PSA declines were confirmed with a subsequent PSA value 2 or more weeks later.

### Data Analysis

The primary objective was to validate that AR-V7–negative patients have prolonged PFS with abiraterone or enzalutamide compared with AR-V7–positive patients at the trial level. OS and response rates (PSA and radiographic) were secondary clinical outcomes. The null hypothesis was that the hazard ratio (HR) of PFS in the two groups is 1.0 versus the alternative hypothesis that the HR of AR-V7–positive to AR-V7–negative patients is 2.0. With 90 projected PFS events, the log-rank test has 85% power to detect an HR of 2.0, equivalent to an improvement in the median PFS of 3 *v* 6 months. The following assumptions were made: binary AR-V7 status (positive and negative), a prevalence of AR-V7 positivity of 30%, and that PFS would follow an exponential distribution. In addition, the log-rank test had 80% power to detect an HR of 2.68 if the true prevalence of AR-V7 positivity was 10% or greater.

Patients with no evaluable CTCs were considered AR-V7 negative, and all patients with sufficient blood collection were analyzed regardless of their evaluable CTCs. In secondary analyses, the proportional hazards model was used for assessing the prognostic value of AR-V7 status for PFS and OS adjusted for Halabi et al^28^ prognostic factors (risk score), including PSA level, alkaline phosphatase, lactate dehydrogenase, opioid analgesic use, Eastern Cooperative Oncology Group performance status, albumin, hemoglobin, and metastatic site (visceral, bone, node only). The Kaplan-Meier method was used to estimate median PFS and OS distributions by AR-V7 status. As a secondary biomarker analysis, logistic regression and proportional hazards models were used to test for the prognostic significance of a high CTC heterogeneity score (defined as a Shannon index ≥ 1.5) in predicting a 50% or greater PSA decline, objective response, PFS, and OS.

## RESULTS

Between May 2015 and January 2017, we enrolled 118 men with high-risk mCRPC from five academic medical centers (Data Supplement). Baseline characteristics of the cohort are listed in Table 1 and the Data Supplement.

Of the enrolled men, 55 were treated with abiraterone, 58 were treated with enzalutamide, and five received both therapies concurrently. With no anticipated or observed differences in outcome (PFS or OS) between therapies, results were combined for the primary analysis (Data Supplement). The median follow-up time among surviving patients was 19.6 months, with 102 PFS events and 53 deaths as of the cutoff date of April 9, 2018, when the clinical database was locked for the primary end point. Median PFS was 5.8 months (95% CI, 4.1 to 7.6 months), and median OS was 20.3 months (95% CI, 17.0 to 27.2 months) for the overall cohort, which reflects the high-risk features of this population.

### AR-V7 Testing

At baseline, 28 men (24%) were AR-V7 positive, 88 (75%) were AR-V7 negative, and two (1%) were unevaluable by the JHU mRNA assay. By comparison, 11 men (9%) were AR-V7 positive, whereas 96 (82%) were AR-V7 negative, and 11 (9%) were unevaluable by the Epic protein-based assay (Data Supplement). The percentage agreement between the two CTC AR-V7 assays was 82% (86 of 105). Most discordant results (17 of 19) were JHU AR-V7 positive but Epic AR-V7 negative (Data Supplement). AR-V7 detection at baseline differed by assay and according to disease burden, clinical prognostic factors, and prior therapy (Data Supplement).

### AR-V7 and Efficacy Prediction

**FIG 1.**
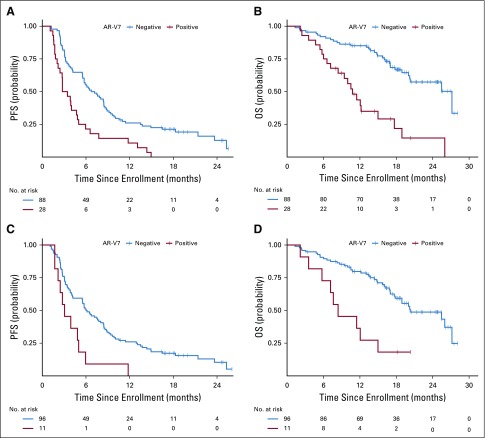
Kaplan-Meier plots of (A) progression-free survival (PFS) and (B) overall survival (OS) by Johns Hopkins University circulating tumor cell androgen receptor splice variant 7 (AR-V7) detection criteria and of (C) PFS and (D) OS by Epic Sciences circulating tumor cell AR-V7 detection criteria.

**FIG 2.**
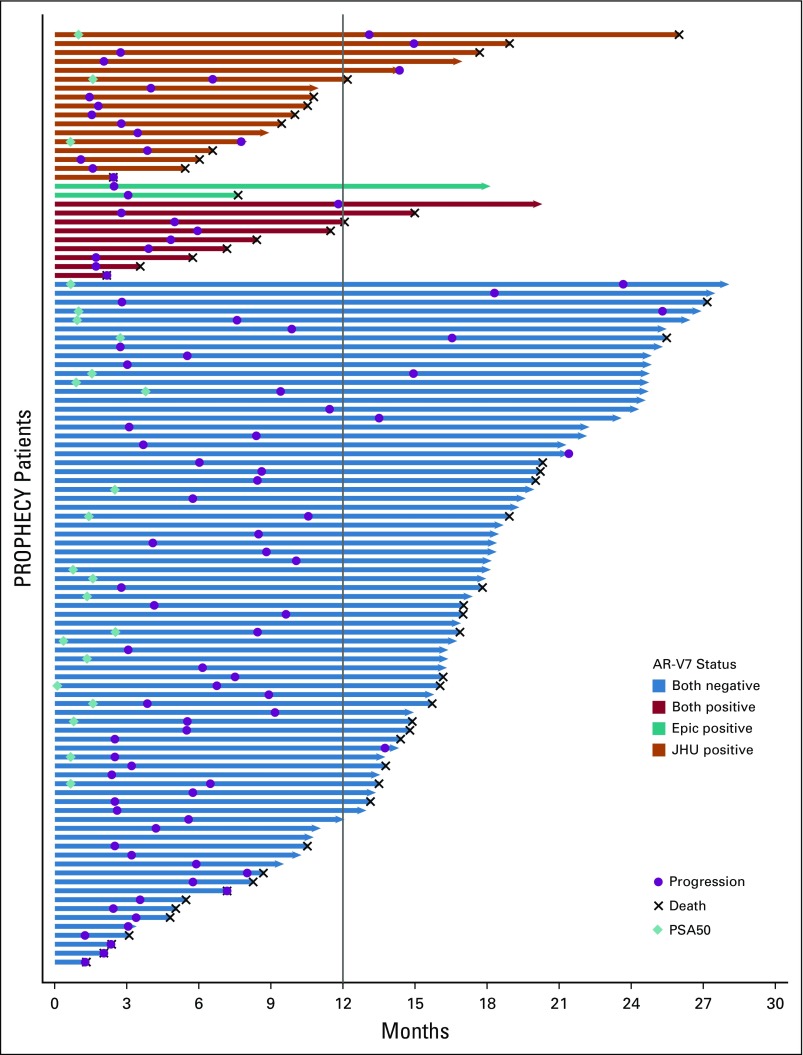
Swimmer plot of patient status according to androgen receptor splice variant 7 (AR-V7) status. Each lane is color coded according to whether the patient tested positive for each AR-V7 test, one test, or neither test or was not evaluable for either test. Epic, Epic Sciences circulating tumor cell AR-V7 protein assay; JHU, Johns Hopkins University circulating tumor cell AR-V7 mRNA assay; PROPHECY, Prospective Circulating Prostate Cancer Predictors in Higher Risk mCRPC [metastatic castration-resistant prostate cancer] Study; PSA50, 50% or greater prostate-specific antigen.

The primary end point of PFS was significantly different in AR-V7–positive men with mCRPC compared with AR-V7–negative men for both AR-V7 assays. For the JHU AR-V7 assay, the median PFS for AR-V7–positive versus AR-V7–negative patients was 3.1 *v* 6.9 months, respectively (HR, 2.4; 95% CI, 1.5 to 3.7). For the Epic AR-V7 protein assay, the median PFS for AR-V7–positive versus AR-V7–negative patients was 3.1 *v* 6.1 months, respectively (HR, 2.5; 95% CI, 1.3 to 4.7). OS differed widely according to AR-V7 status. For the JHU AR-V7 mRNA assay, median OS for AR-V7–positive versus AR-V7–negative patients was 10.8 *v* 27.2 months, respectively (HR, 3.9; 95% CI, 2.2 to 6.9). For the Epic AR-V7 protein assay, the median OS for AR-V7–positive versus AR-V7–negative patients was 8.4 *v* 25.5 months, respectively (HR, 3.4; 95% CI, 1.6 to 7.0). Table 2 lists and Figure 1 shows the results of PFS and OS by baseline AR-V7 status for each CTC assay. Figure 2 shows a swimmer plot of each patient’s experience according to CTC AR-V7 baseline status, which demonstrates shortened times to progression and OS for AR-V7–positive versus AR-V–negative men according to each assay. Results stratified by three categories (CTC negative, CTC positive, and AR-V7 positive or negative) are shown in the Data Supplement.

**TABLE 2.**
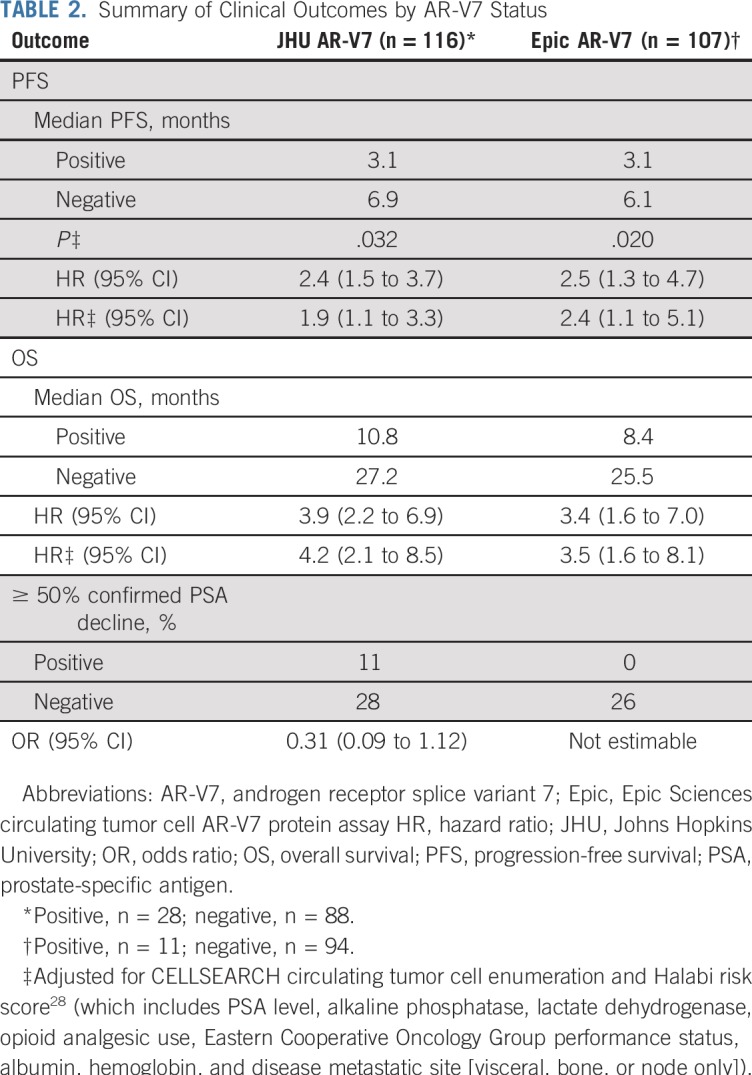
Summary of Clinical Outcomes by AR-V7 Status

In a multivariable analysis of baseline AR-V7 status, adjustment for baseline CTC enumeration (CELLSEARCH), Halabi prognostic risk score,^28^ and CTC AR-V7 by the JHU assay was significantly associated with worse PFS (adjusted HR, 1.9; 95% CI, 1.1 to 3.3; *P* = .032). The Epic AR-V7 assay also was associated with worse PFS (adjusted HR, 2.4; 95% CI, 1.1 to 5.1; *P* = .020; Data Supplement). Halabi risk score and CELLSEARCH CTC enumeration were not associated with PFS after adjustment for AR-V7 status.

CTC AR-V7 detection by both assays was independently associated with worse OS in multivariable analysis. The HRs for death for AR-V7 positivity were 4.2 (95% CI, 2.1 to 8.5) and 3.5 (95% CI, 1.6 to 8.1) for the JHU and Epic assays, respectively (Data Supplement). Halabi risk score and CELLSEARCH CTC enumeration were not associated with OS after adjustment for AR-V7 status, but risk score was associated with PFS and OS in JHU AR-V7–negative patients (Data Supplement). OS was 12 or fewer months after abiraterone or enzalutamide initiation for 63% of Epic AR-V7–positive men (seven of 11), 54% of JHU AR-V7–positive men (15 of 28), 47% of JHU AR-V7–positive and Epic AR-V7–negative men (eight of 17), and 14% of AR-V7–negative men by either test (11 of 77).

**FIG 3.**
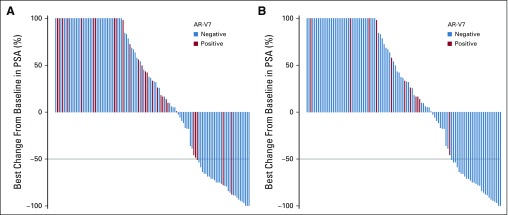
Prostate-specific antigen (PSA) waterfall plots of the best overall confirmed PSA decline from baseline with abiraterone or enzalutamide according to (A) Johns Hopkins University circulating tumor cell androgen receptor splice variant 7 (AR-V7) status and (B) Epic Sciences circulating tumor cell AR-V7 status.

For the key secondary outcomes of best overall confirmed PSA decline or soft tissue responses with abiraterone or enzalutamide treatment, no Epic AR-V7–positive patients had a confirmed PSA or RECIST response. Eleven percent of JHU AR-V7–positive patients had a confirmed PSA response, and 6% (three of 51) had a RECIST response. By comparison, there were 26% to 28% with confirmed PSA responses and 21% to 25% with soft tissue responses in AR-V7–negative patients by either assay (Fig 3). PSA declines were associated with improved PFS and OS in AR-V7–negative men (Data Supplement).

Finally, we examined the relationship of AR-V7 heterogeneity within patients and over time and the relationship of AR-V7 to high CTC phenotypic heterogeneity denoted by an elevated Shannon index.^22^ A high Shannon index was associated with poor OS (median OS, 11.5 *v* 25.5 months; HR, 2.3; 95% CI, 1.3 to 4.2) and poor PFS (median PFS, 4.0 *v* 6.5 months; HR, 1.8; 95% CI, 1.1 to 3.1) in univariable analysis and a lower proportion of confirmed PSA responses (11% *v* 26%; Data Supplement). Epic AR-V7–positive patients were more likely to have high CTC heterogeneity previously (Shannon index ≥ 1.5), which indicates a higher diversity of cellular phenotypes. Sixty-four percent of AR-V7–positive men (seven of 11) had high heterogeneity versus only 14% (13 of 96) of AR-V–negative men. AR-V7 positivity by the JHU assay was independently associated with PFS (HR, 1.91; 95% CI, 1.12 to 3.26; *P* = .017) and OS (HR, 3.90; 95% CI, 2.02 to 7.56; *P* < .001) after adjusting for CTC number and Shannon index, whereas the Shannon index and CTC number were not significantly associated with PFS or OS (*P* not significant). Epic AR-V7 positivity also was associated with PFS (HR, 2.63; 95% CI, 1.17 to 5.93; *P* = .019) and OS (HR, 3.61; 95% CI, 1.64 to 7.93; *P* < .001), whereas CTC number and Shannon index were not associated with either PFS or OS (*P* not significant).

**FIG 4.**
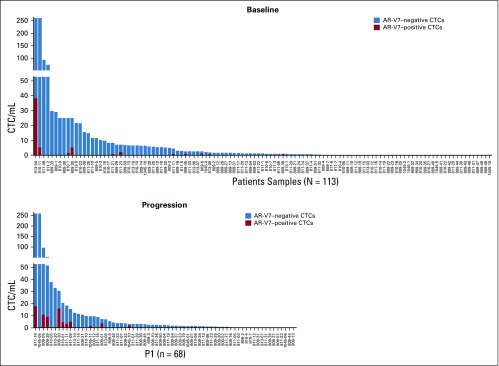
Plot of the proportion of circulating tumor cells (CTCs) that tested positive for androgen receptor splice variant 7 (AR-V7) nuclear protein (red) at (A) baseline and at (B) progression on abiraterone acetate or enzalutamide as a function of the total number of CTCs, including AR-V7–negative CTCs (blue).

Finally, although the majority of CTCs in men with mCRPC were AR-V7 negative, even in AR-V7–positive patients, the proportion of AR-V7–positive cells ranged from 1% to 100% (median, 20%; Fig 4). At progression on abiraterone or enzalutamide, 14 (20%) of 69 evaluable men had AR-V7 detection by Epic criteria, and 26 (34%) of 77 evaluable men had AR-V7 detection by JHU criteria, which suggests the induction or selection of AR-V7 expression.

## DISCUSSION

Few biomarkers used in cancer care undergo independent, prospective testing for their predictive or prognostic significance, which results in a paucity of validated tools to inform patient care. On the basis of single institutional testing, AR-V7 represents one of the most specific, novel, and promising markers to guide treatment decisions in patients with poor prognosis mCRPC^13-16,24,29^ when selection of an effective treatment is critical to maximizing quality of life and survival. In this prospective, multicenter, blinded study of AR-V7 detection in CTCs, we demonstrate that men with high-risk mCRPC who are AR-V7 positive by either of two different assays have little evidence of clinical benefit from abiraterone or enzalutamide, a very low probability of confirmed PSA decline, and a short OS and PFS.

In this study cohort, approximately 10% to 24% of men with high-risk mCRPC were AR-V7 positive at baseline, depending on the assay used. The proportion of AR-V7–positive men with a confirmed PSA decline or radiographic response with abiraterone or enzalutamide ranged from 0% to 11%; the majority of such men had progressive disease, with PFS estimates of approximately 3 months in most cases. Although a minority of men who harbored AR-V7 had a 50% or greater decline in PSA from baseline, PFS times were generally short, with only two (7%) of 28 JHU AR-V7–positive men having PFS times of more than 12 months and only one (9%) of 11 Epic AR-V7–positive men having a PFS time of more than 6 months. Thus, knowledge of the AR-V7 status using one of these blood-based assays, in conjunction with standard clinical prognostic measures, may predict the probability of benefit from abiraterone or enzalutamide.

Our findings suggest that although a positive AR-V7 test is associated strongly with hormonal resistance, high-risk AR-V7–negative men may still not respond to AR inhibition, despite a greater probability of response. Alternative resistance mechanisms independent of AR-V7 include lineage plasticity and AR indifference,^30,31^ glucocorticoid receptor activation,^32^ AR gain or ligand binding domain mutations,^33-35^ alternative AR variants and genomic structural rearrangements,^36-38^ AR enhancer amplification,^39^ and additional compensatory oncogenic pathways.^33^ PSA declines with abiraterone or enzalutamide are associated with improved PFS and OS, which supports PSA monitoring for AR-V7–negative men with mCRPC.^40^ AR-V7 likely explains up to 25% of AR therapy resistance, which implies that most treatment resistance mechanisms remain unidentified. Although the JHU assay resulted in more AR-V7–positive patients versus the Epic assay (24% *v* 9%), these differences may relate to CTC detection differences between assays and the greater sensitivity needs of nuclear AR-V7 protein detection. Despite this, men who tested positive by the JHU AR-V7 assay were confirmed to have poor outcomes and a low probability of response to abiraterone or enzalutamide. Trade-offs between assay sensitivity and specificity for the prediction of response to abiraterone or enzalutamide are clearly present, with the Epic assay providing no false-positive results and the JHU assay detecting twice as many AR-V7–positive men but resulting in 6% to 11% of AR-V7–positive men with confirmed PSA/radiographic responses. Critical to the development of a precision medicine algorithm for men with mCRPC will be the standardization and clinical validation of assays that capture novel mechanisms in a timely manner for consideration in treatment decisions. Our data support AR-V7 as one such important prognostic biomarker for mCRPC.

An objective measure of cellular heterogeneity using the Epic platform (Shannon index)^22^ demonstrated a direct correlation between heterogeneity and AR-V7 positivity, which suggests greater tumor cell entropy and inherent resistance to AR targeting in these patients. Of note, we find that the negative prognostic and predictive significance of AR-V7 testing using either assay was independent of the number of CTCs,^17,20^ CTC heterogeneity, and other clinical prognostic measures of disease burden,^18,41,42^ which support the hypothesis that AR-V7 may be causally related to these poor outcomes and treatment resistance. These results, coupled with preclinical mechanistic studies demonstrating ligand-independent activation of AR-V7 in promoting the AR transcriptional program and treatment resistance^11,12^ and the increased detection of AR-V7 in some progressing patients on AR inhibiting therapies,^9^ support the concept that AR-V7 is associated with both phenotypic heterogeneity and AR therapy resistance. Trials are still needed to address whether AR-V7 is a driver of disease resistance through therapeutic targeting of AR N-terminal or DNA-binding domains. Our data suggest that AR-V7 is highly associated with rapid resistance to hormonal therapy and disease heterogeneity and is enriched at progression during treatment with AR inhibitors even after adjusting for disease burden and CTC enumeration.

One limitation of our study is the lack of testing with alternative treatment strategies in AR-V7–positive men with mCRPC, such as docetaxel chemotherapy. However, prior work suggests that AR-V7 positivity by either assay is adversely prognostic in men with mCRPC but is associated with better outcomes and response to taxane chemotherapy compared with poor outcomes with AR inhibitors in this population.^14-16^ Testing patients with multiple poor-risk prognostic features similar to those included in our study could therefore inform the decision to proceed with hormonal therapy or docetaxel chemotherapy. AR-V7–positive men with mCRPC still have a reasonable probability of response and clinical benefit with chemotherapy.^15^ Hence, the current results will inform clinical practice given the confirmed low probability of benefit with current AR inhibitors in AR-V7–positive men, particularly in those previously exposed to potent AR inhibitors. A recent study that evaluated the real-world clinical utility of AR-V7 testing suggested a therapeutic benefit using a biomarker-informed (rather than a biomarker-agnostic) approach in the management of mCRPC.^25^ A second limitation is the inclusion of only poor-risk men who were more likely to have CTCs and informative results; men with no CTCs and a more favorable prognosis, particularly in the first-line setting, will likely test negative for AR-V7 and may not benefit from AR-V7 testing.^24^ Finally, although our sample size was sufficient for independent multivariable prognostic validation of AR-V7, there were only 11 and 28 men who tested positive for AR-V7 by the Epic and JHU assays, respectively, which limits power for broader multivariable analyses. Larger controlled studies that more comprehensively assess CRPC genotypes, phenotypes, and AR splice variants are needed to confirm the predictive value of AR-V7 in the context of the host and CRPC genomic factors.

In conclusion, we have prospectively demonstrated that AR-V7 is a strong predictor of clinical outcomes in men with mCRPC treated with abiraterone or enzalutamide. In patients with multiple clinical indicators of poor prognosis, we can identify a significant subset of patients with detectable AR-V7 by two independent assays. Knowledge of AR-V7 status may optimize treatment selection beyond clinical measures of prognosis and disease burden. The PROPHECY study represents a multicenter effort that provides prospective, blinded clinical validation around such an approach and suggests that both the JHU CTC AR-V7 mRNA assay and the Epic CTC nuclear-specific AR-V7 protein assay provide clinical utility around the anticipated outcomes with therapy.
